# The Developmental Progression of Early Algebraic Thinking of Elementary School Students

**DOI:** 10.3390/jintelligence11120222

**Published:** 2023-12-06

**Authors:** Siyu Sun, Dandan Sun, Tianshu Xu

**Affiliations:** 1 College of Education, Capital Normal University, Beijing 100048, China; 2 School of Mathematics and Statistics, Shandong Normal University, Jinan 250358, China; ddsun@sdnu.edu.cn; 3 School of Educational Science, Nantong University, Nantong 226019, China; tshxu@ntu.edu.cn

**Keywords:** early algebra, algebraic thinking, elementary school student, latent class analysis, generalized arithmetic, functional thinking, quantitative reasoning

## Abstract

Developing algebraic thinking in elementary school has gained consensus among mathematics educators. The objective of this study is to understand the developmental trajectory of early algebraic thinking in elementary school students so as to assist teachers and curriculum developers in implementing instruction that aligns with students’ cognitive development. This study adopted a cross-sectional survey approach, involving 526 students from grades three to five in Shanghai, who were tested using a 12-item assessment that measured three aspects: “generalized arithmetic”, “functional thinking”, and “quantitative reasoning”. Latent class analysis was used to analyze students’ response strategies, and, in conjunction with individual interviews, this study identified potential developmental pathways in students’ early algebraic thinking, progressing from “arithmetic thinking” to “concrete algebraic thinking”, “generalized algebraic thinking”, and finally to “symbolic algebraic thinking”. As thinking levels advanced, significant differences in students’ response strategies emerged, with notable improvements in “generalization abilities” and “symbolization abilities”. This study suggests that educational practices should encompass content in elementary arithmetic curricula that fosters generalization abilities. Additionally, providing students with opportunities for diverse representations can effectively stimulate the development of early algebraic thinking.

## 1. Introduction

Algebraic ability serves as a critical factor in the academic and cognitive development of students. Existing research indicates that such competence has significant implications not only for university admissions but also for long-term career prospects, influencing the range and quality of employment opportunities available to individuals (Moses and Cobb 2002; Attewell and Domina 2008). Additionally, acquiring foundational algebraic knowledge and skills, as well as cultivating an initial level of algebraic reasoning, serve as crucial prerequisites for the in-depth exploration of more advanced mathematical topics (Schoenfeld 1995). Over the past two decades, as research on algebraic teaching and learning has deepened, scholars in the field of mathematics education have increasingly coalesced around a shared understanding: The cultivation of algebraic thinking ought to commence in elementary school and continue throughout the entire trajectory of mathematics education (NCTM 2000; Stacey et al. 2004). A great number of studies have confirmed that elementary school students can develop algebraic thinking and grasp related algebraic concepts (Schifter 1999; Wilkie and Clarke 2016; Irwin and Britt 2005). Veith et al. (2023) identified early algebra to be the very motor theme of algebra education research.

As research into elementary school algebra teaching and learning continues to expand, early algebra has emerged as a distinct field of study. Initial investigations primarily concentrated on delineating pathways for fostering algebraic thinking in primary school students, with various scholars proposing divergent strategies. For instance, Davydov centered his curriculum around the core concept of quantitative reasoning (Lee 2002). Numerous other scholars have underscored the pivotal role that relational thinking plays in nurturing algebraic reasoning among elementary school students (Stephens 2006; Carpenter et al. 2005). Additionally, Blanton and colleagues have dedicated their work to cultivating functional thinking in primary school students (Blanton and Kaput 2011). Most of these studies have created curricula aimed at facilitating the development of algebraic thinking in elementary school students (Dougherty 2008; Lee 2002). While some research has been directed toward understanding elementary students’ comprehension of the equal sign (Blanton 2013) and functional thinking (Blanton et al. 2015b; Stephens et al. 2017b), the scope of the test content in these studies remains limited. There is a notable scarcity of quantitative research offering a comprehensive examination of elementary school students’ early levels of algebraic thinking.

Conducting such an investigation into students’ levels and developmental trajectories in algebraic thinking is both necessary and valuable. This information could greatly assist curriculum developers in crafting educational courses that are better aligned with the evolving cognitive pathways of students. Research has shown that the Chinese elementary school mathematics curriculum has an advantage in fostering early algebraic thinking (Cai 2004a). Additionally, the outstanding mathematics performance of Chinese students in large-scale international assessments like PISA has garnered attention from mathematics researchers (OECD 2020). Currently, research on early algebra in China primarily focuses on textbook analysis (Ding et al. 2023) and curriculum comparisons (Cai et al. 2011), with relatively less attention given to students’ developmental levels of thinking. There is a shortage of research, especially regarding the exploration of developmental pathways and levels of algebraic thinking. Therefore, conducting a quantitative investigation into the developmental pathways of early algebraic thinking among Chinese elementary students is beneficial for enhancing our understanding of students’ cognitive development, allowing teachers to tailor instruction to individual students and implement personalized teaching strategies.

Based on the above discussion, we administered a test focused on generalized arithmetic, functional thinking, and quantitative reasoning to 526 Chinese students across grades 3–5. Through an analysis of students’ problem-solving strategies, this study seeks to address the following research questions:

(i). Do distinct groups emerge, characterized by different developmental states, among students based on their performance in the early algebraic thinking test?
(ii). What specific characteristics of early algebraic thinking can be observed among these various student groups?
(iii). Do these observed differences suggest a developmental trajectory in early algebraic thinking, transitioning from more intuitive forms to more sophisticated ones?

## 2. Theoretical Framework

### 2.1. The Key Components of Early Algebraic Thinking

Traditionally, equations are typically present in the initial stage of students’ algebra learning. However, as research in early algebra education has evolved, scholars have come to understand that while the use of expressions and alphabetic representation for numbers are indeed important facets of algebraic instruction, they are not necessarily the singular, nor the optimal, entry points for students’ algebraic learning. The leap that students make from engaging solely with numerical concepts to navigating a landscape filled with algebraic symbols is a transition often underestimated or overlooked. Therefore, the kind of algebra view that is manipulated by symbols is quite narrow. Algebra is not just some static knowledge but a mathematical activity that involves behaviors such as speculation, induction, representation, proof, and communication (Kaput 2008). This dynamic perspective on algebraic thinking has also garnered acknowledgment from other scholars in the field. For instance, Kieran et al. (2016) contended that current research in early algebra predominantly concentrates on the reasoning processes employed by children aged 6–12 to establish mathematical relationships, patterns, and arithmetic structures. These processes include attentiveness, conjecturing, generalization, representation, and argumentation. Based on this dynamic view of algebra, many researchers have begun to explore the possibility of developing students’ algebraic thinking in elementary school.

Earlier, Kaput (2008) conducted systematic research on early algebra and identified “generalization” as the core of early algebraic thinking, pointing out three algebra core content strands: (i) generalized arithmetic, (ii) functional thinking, and (iii) the application of generalizations as modeling languages. Many studies are based on Kaput’s theory, using a similar framework to investigate students’ early algebraic thinking (Ralston et al. 2018; Blanton et al. 2015a; Chimoni et al. 2018). Similarly, Stephens et al. (2017a) offered a comprehensive review of research in the realm of early algebraic thinking. They note that contemporary studies predominantly concentrate on three key mathematical domains: generalized arithmetic, functional thinking, and quantitative reasoning. These studies also pay attention to four critical cognitive processes: generalization, reasoning, representation, and justification.

Based on previous studies (Kaput 2008; Kieran et al. 2016; Stephens et al. 2017a), this study defines “early algebraic thinking” as a series of cognitive processes that children undergo when inductively summarizing and generalizing the structure, patterns, and quantitative reasoning in mathematical formulas. Additionally, this includes the utilization of symbolic representation to articulate and logically substantiate generalized conclusions. The crux of the matter lies in the development of students’ skills in generalization, cultivated through the process of arithmetic learning. This is principally evident in their engagement with generalized arithmetic, functional thinking, and quantitative reasoning. “Generalized arithmetic” primarily refers to students’ ability to understand equations and identify and generalize the underlying structures in mathematical expressions. This includes understanding the fundamental properties and laws of operations, as well as the arithmetic relationships of specific types of numbers, such as the relationships involved in the arithmetic of even and odd numbers. “Functional thinking” refers to students’ ability to understand variables, covariation, and correspondence relationships. Additionally, students are capable of summarizing, representing, and reasoning about the relationships between variables using natural language, algebraic symbols, drawings, tables, and charts. “Quantitative reasoning” refers to the process of establishing quantitative relationships between known and unknown quantities in the context of mathematical problems, and then reasoning about the relationships among these quantities.

These three concepts exhibit both differences and close connections. Generalized arithmetic emphasizes summarizing operational rules in arithmetic contexts, while functional relationships primarily involve summarizing the correspondence between independent and dependent variables. Quantity reasoning, on the other hand, generalizes the equivalence relationships among different quantities. The common core of these three concepts is the cultivation of students’ generalization abilities, albeit originating from different mathematical contexts. They all serve as entry points for developing students’ algebraic thinking in elementary arithmetic courses and play a significant role in nurturing such thinking. The importance of these concepts extends beyond elementary education and holds significant value in tertiary education as well (Veith and Bitzenbauer 2022).

### 2.2. Levels of Students’ Thinking about Early Algebraic Concepts

Prior research has explored the various content domains within students’ early algebraic thinking, revealing that the evolution of mathematical cognition occurs in distinct phases. For example, understanding equality and equations is an important aspect of generalized arithmetic. Rittle-Johnson et al. (2011) conducted a survey on elementary school students’ understanding of equations and found that their thinking levels showed a developmental path from rigid operational to flexible operational, to basic relational, and ultimately to comparative relational. Similarly, Blanton (2013) has also categorized students’ understanding of the equal sign into operational thinking, computational thinking, and relational thinking through systematic investigations. Specifically, students in operational thinking can only understand the equal sign as a sign of output as a result. Therefore, when students answer the mathematical question 5 + 7 = __ + 3, they will fill in the blank with 12 (because 5 + 7 = 12), resulting in the incorrect Equation 5 + 7 = 12 + 3. Students at the computational thinking stage can discern that 5 + 7 and 9 + 3 are equivalent by recognizing that both expressions yield a sum of 12, thereby concluding that 5 + 7 = 9 + 3. Students at the relational thinking stage can comprehend that 5 + 7 equals 9 + 3 by analyzing the relationship between the numbers, noting that 3 is 2 less than 5 and 9 is 2 more than 7.

Functional thinking is an important component of algebraic thinking. As research in early algebraic thinking continues to evolve, numerous scholars have highlighted that the onset of algebraic reasoning is not confined to solving equations. Instead, it is rooted in the establishment and manipulation of relationships, particularly those of a functional nature (Stephens et al. 2017b; Warren et al. 2006). Studies have shown that elementary school students have the ability to generalize and characterize functional relationships (Tannisli 2011; Greens and Rubenstein 2008; Wilkie and Clarke 2016), even among lower-grade students (Blanton et al. 2017; Molina et al. 2018). Some studies found that students initially focused on recursive patterns of dependent variables, rather than relationships between varying quantities (Warren et al. 2006; Lannin et al. 2006). However, under effective teaching guidance, elementary school students can develop covariational thinking (discovering the covariation relationship between two variables) and even correspondence thinking (discovering the functional rules between two variables) (Stephens et al. 2017b; Thompson and Carlson 2017).

Although it is still a controversial topic whether elementary school students are required to use letters to represent numbers (Drouhard and Teppo 2004), the important role of representation skills in early algebraic thinking is self-evident (Radford 2014). Blanton et al. held qualitative interviews with first-grade students, focusing on tasks related to functional thinking. They categorized the students’ understanding of variables into six distinct levels: pre-symbolic understanding, letters as labels, fixed and deterministic values, fixed but arbitrary values, unknowns, and mathematical objects (Blanton et al. 2017). This indicates that students already have their own understanding of variables before learning how to represent numbers with letters in the classroom. Certainly, the roles that letters can play in representing numbers are multifaceted (Usiskin 1988), and it is worth noting that even high school and college students may exhibit underdeveloped understanding in some of these functional areas (Brizuela et al. 2015; Reisch 2008). Generalization is the core of early algebraic thinking. Thus, for elementary school students, the use of informal representations like natural language, graphical illustrations, and tables should be actively encouraged. These formats are conducive to fostering the advancement of students’ algebraic thought processes (Radford 2018).

## 3. Materials and Methods

### 3.1. Procedure and Participants

The aim of this research is to investigate the early algebraic thinking of elementary school students and to discern potential developmental trajectories. A cross-sectional approach was employed, which involves selecting samples from various developmental stages concurrently (Cohen et al. 2007). Drawing on expert recommendations and literature review, this study focuses on students from Grade 3 to Grade 5, aged from 8 years old to 10 years old (with Grades 1 through 5 constituting primary education in Shanghai) for three primary reasons. Firstly, a textbook analysis reveals that content related to algebraic thinking starts in Grade 3, ensuring that students from this grade onward possess the foundational skills required for algebraic assessment, which has also been confirmed by the recent study conducted by Ding et al. (2023). Secondly, abstract thinking and the capacity for generalization begin to manifest in students around the third grade. Lastly, students in Grade 3 exhibit enhanced writing and verbal expression skills, equipping them to articulate their reasoning in response to test questions. Additionally, in this research, we employed case-based interviews. The first author conducted semi-structured discussions with students exhibiting characteristic response strategies, aiming to delve deeper into their thought processes.

The participants were a convenience sample consisting of 526 students. This sample consisted of 190 third-graders, 161 fourth-graders, and 175 fifth-graders. These students hail from two public primary schools in Shanghai: one located downtown and the other in the suburbs. Mathematics teachers in each class administered the test, which had a duration of 45 min. Prior to the testing, examiners underwent formal training in test administration and response documentation procedures. After the testing, researchers collected and analyzed all completed test papers.

### 3.2. Instruments

The Early Algebraic Thinking Test (EATT) consisted of 12 items that were adapted from previous research studies (Rittle-Johnson et al. 2011; Ralston et al. 2018; Blanton et al. 2015a; Chimoni et al. 2018; Kolovou et al. 2013), which contained the three core algebra content strands. Specifically, six of the items are generalized arithmetic tasks. Three modeling tasks and three quantitative reasoning tasks constitute the remaining six items. The specific contents strand and concepts of items in EATT12 are shown in Table 1.

Generalized arithmetic tasks refer to the concepts of equal sign, the manipulation of operations and their properties, and the transformation and solution of equations. Tasks GA1 and GA2 (adapted from Rittle-Johnson et al. 2011) required students to determine whether the equation was true and find the missing quantity in equations relationally (e.g., GA1: Can you determine whether this equation is true or false without calculating? 67 + 86 = 68 + 85). Tasks GA3 and GA4 (adapted from Chimoni et al. 2018) presented an equation that included numbers and symbols; students were expected to calculate the value of the symbols (GA3: ☆ + 12 = ☆ + ☆ + ☆, ☆ = ___; GA4: If ⚪ + ⚪ = 16, then ⚪ + ⚪ + 5 = ____). In Task GA5 (adapted from Irwin and Britt 2005), students had to imitate the strategies in the topic to complete the simple calculation, which means the ability to quickly and accurately solve basic mathematical operations such as addition, subtraction, multiplication, and division. Task GA6 (adapted from Zhu 2017) required students to notice the structure of the expressions, expand two more similar expressions, and use symbols to present the property of operation.

**Figure 1:**
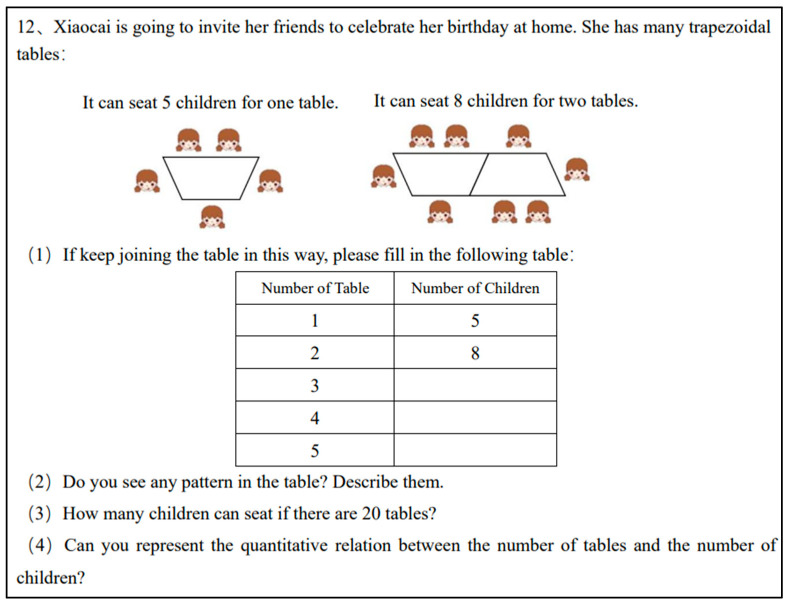
Functional thinking Task FT12.

Functional thinking tasks involve the generalization of relationships between covarying quantities. These tasks are related to the ability to express numerical and figure patterns as functions and algebraic expressions. Tasks FT7 and FT12 (adapted from Ralston et al. 2018; Blanton et al. 2015b) represented the figural pattern and asked for a next term and a distant term (e.g., Figure 1). In Task FT11 (adapted from Blanton et al. 2015a), students were required to identify a variable to represent an unknown quantity and algebraic expression.

Quantitative reasoning tasks involve the generalization of quantitative relationships that are presented implicitly through various problem contexts. In Tasks QR8, QR9, and QR10 (adapted from Kolovou et al. 2013), students had to recognize the quantitative relationship in the word problems and use letters to represent the relationship.

### 3.3. Coding Scheme

The coding scheme was further developed as student responses were examined and unanticipated responses were encountered. When new codes were introduced, their meanings were negotiated among the coders. Inter-rater reliability was established using a randomly selected sample comprising 40% of responses. The first author and the third author were involved in the coding work for the rating agreement. Any discrepancies were discussed until a full agreement was reached.

Apart from the objective multiple-choice questions on quantitative reasoning (QR8, QR9, and QR10), which are scored as either 0 or 1, the other questions in this test employ a graded coding system, awarding scores ranging from 0 to 3 or 0 to 4 based on students’ answering strategies. The Cronbach’s α values ranged from 0.924*** to 1.000***. For example, for GA1 (Can you determine if this equation is true or false without calculating? 67 + 86 = 68 + 85), children with computational thinking can only determine equality by calculating 67 + 86 = 153 and 68 + 85 = 153. However, children with relational thinking can deduce the equation’s equality by noting that “68 is 1 more than 67, while 85 is 1 less than 86”. Therefore, this question is graded into four levels: a blank answer or selecting “false” in (1) receives 0 points; selecting “true” in (1) but leaving the reason blank or providing a wrong reason receives 1 point; selecting “true” in (1) and providing the reasoning through calculation (i.e., 67 + 86 = 153, 68 + 85 = 153) receives 2 points; and deducing the equation’s equality through relational thinking receives 3 points. Generalized arithmetic tasks are graded based on similar criteria.

Questions 7, 11, and 12 were designed to assess students’ functional thinking skills. Functional thinking predominantly involves identifying the relationship between the independent and dependent variables in problems that exhibit patterns. According to Stephens et al. (2017b), their functional thinking can be analyzed through generalization and different types of functional thinking processes, which are recursive, covariation, and correspondence. Using Question 12 (illustrated in Figure 1) as an example, a blank or entirely incorrect answer earns 0 points; if only part (1) is answered correctly, it scores 1 point. If part (2) identifies the correct pattern (e.g., recursive pattern, covariation pattern, or correspondence pattern), it scores 2 points. Correctly determining the number of children for the 20th table earns 3 points. Lastly, if one can generalize the relationship between the number of tables and the number of children using an algebraic expression, they score 4 points.

Apart from the grading criteria for the 12 tasks, we also categorized student’s types of functional thinking and modes of representation, based on the holistic review of their responses. The modes of representation are ranked from 0 to 3, denoting, in order, no representation, representation using natural language, representation via diagrams, and representation with symbols. Similarly, the types of functional thinking are rated from 0 to 3, corresponding to the absence of any rule, recursive thinking, covariant thinking, and correspondence thinking.

### 3.4. Data Analysis

To address our research questions, we began our analysis using a latent class analysis (LCA) implemented in Mplus (Muthén and Muthén [1998] 2010). The primary purpose of LCA is to categorize individuals based on various indicators. Individuals within a particular latent class exhibit similar response patterns, whereas those across different classes differ significantly (Weller et al. 2020; Collins and Lanza 2010). Model fit was assessed using the Akaike information criterion (AIC), Bayesian information criterion (BIC), and adjusted Bayesian information criterion (aBIC), where lower values indicate better fit (Lo et al. 2001). In addition, the quality of classification is often evaluated using the entropy criterion, with values close to 1 indicating high classification accuracy. A statistically significant result of the Lo–Mendell–Rubin (LMR) test and the bootstrap likelihood ratio test (BLRT) at a significance level of *p* < .01 suggests that the LCA model with t latent classes is a better fit for the data compared to the t−1 class model (Yu et al. 2017).

In this research, the LCA was employed to discern potential clusters of students from the 526 participants, based on their response patterns to the early algebraic thinking tasks. Analyzing the responses of students within these identified clusters allows for a deeper understanding of their early algebraic thinking. Subsequently, in the second phase of our analysis, we utilized the SPSS software to derive both descriptive and inferential statistics related to student performance. As a complement to the quantitative methods, we qualitatively summarized the thinking characteristics of four categories of students based on their responses to paper-and-pencil tests. This addressed the second research question of our study. In addition, to explain more details of the thought patterns of each type of student, we presented the cases in the form of interviews.

## 4. Results

### 4.1. Identification of Different Groups of Students Based on Their Performance

The latent class analysis (LCA) model was established using maximum likelihood estimation, with the number of classes ranging from 1 to 6. Table 2 represents the model fit statistics.

In Table 2, it is evident that AIC exhibits a decreasing trend as we move from top to bottom, while BIC attains its minimum value when the population is partitioned into three categories. Similarly, aBIC reaches its minimum value when the population is segmented into four categories. Notably, the highest value of the entropy index is recorded when the population is subdivided into six categories. The LMR index loses its significance after partitioning the population into the third category. Conversely, the BLRT index demonstrates that all categorizations prior to the sixth category outperform their respective predecessors.

We did not initially reach a consensus regarding the criteria for model selection, with the final choice primarily dependent on the interpretability of the data. Considering both model fit and classification accuracy, Class 3 and Class 4 were given priority. When students were divided into three categories, the group sizes for these three groups were 247 (50%), 162 (30.8%), and 117 (22.2%), respectively. When students were divided into four categories, the group sizes for these four groups were 28 (5.3%), 67 (12.7%), 220 (41.8%), and 211 (40.1%), respectively. The qualitative analysis of student data classified with the two models revealed that students grouped into four categories exhibited more significant cognitive characteristics, with an entropy value of 0.83, which is higher than that of the three-category designation, at 0.78, indicating higher classification accuracy.

**Figure 2:**
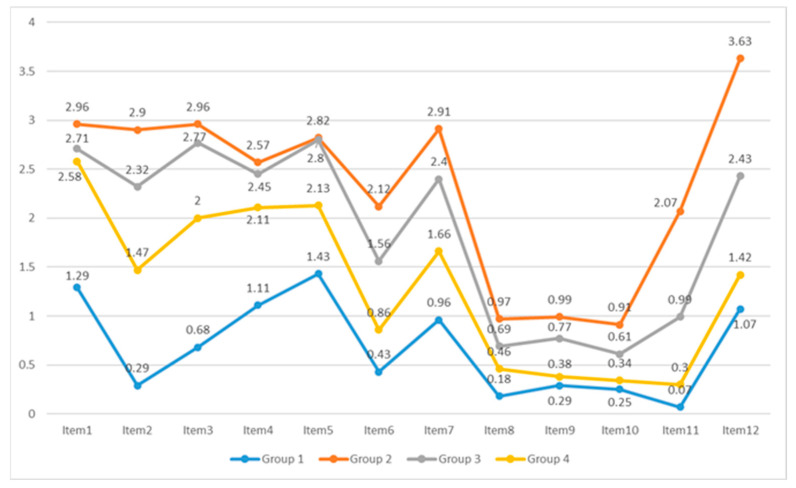
The average scores of four groups of students on 12 mathematical tasks. For the 12th item, a full score is 4, while Items 8 to 10 have a full score of 1, and all other items have a full score of 3.

Considering the fitting parameters from Table 2 and the clarity of student response characteristics, we ultimately categorized students into four groups. Notably, this configuration yielded the minimum aBIC value of 11,516.04, while the entropy value of 0.83 (>0.7) signified an impressive classification accuracy exceeding 80%. Moreover, the statistically significant BLRT confirms that the four-category model outperforms the three-category alternative. Figure 2 depicts the average scores of the four student groups across 12 assessment items. For the 12th item, a full score is 4, while Items 8 to 10 have a full score of 1, and all other items have a full score of 3.

From Figure 2, it can be observed that the second group of students performed the best across the 12 tasks, followed by the third group, while the first group had the lowest average scores and ability values. The first group consisted of 28 students, accounting for 5.3% of the total; the second group included 67 students, constituting 12.7%; the third group was the largest with 220 students, constituting 41.8%; and the last group consisted of 211 students, accounting for 40.1%.

### 4.2. The Characteristics of the Responses Provided by Students in Each Group in Specific Tasks

Through latent class analysis, students were categorized into four groups. After conducting a qualitative analysis of the answer strategies employed by these four student groups across 12 mathematical tasks, these four categories of students were named in ascending order considering cognitive levels as follows: arithmetic thinking (Group 1), concrete algebraic thinking (Group 4), generalized algebraic thinking (Group 3), and symbolic algebraic thinking (Group 2). In the next sections, we will provide an overview of the cognitive traits and typical instances within each of the four student groups.

#### 4.2.1. Arithmetic Thinking Students

**Figure 3:**
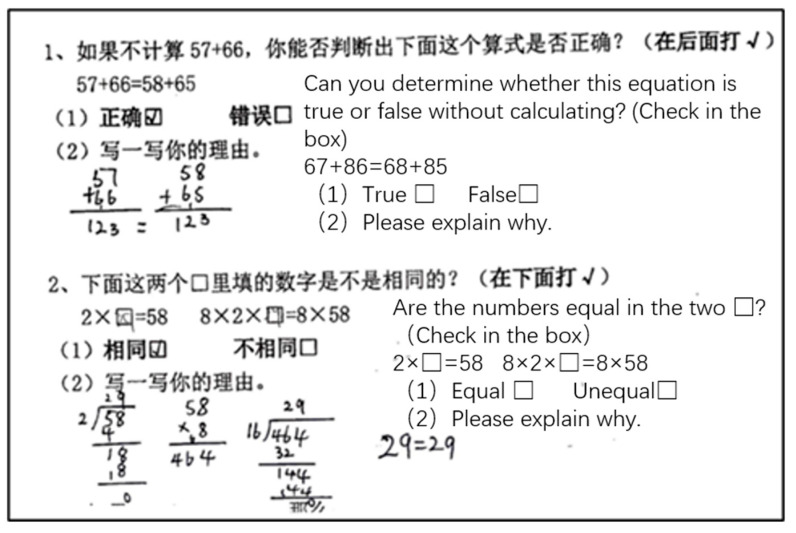
The strategies employed by students with arithmetic thinking in G1 and G2.

One of the distinguishing characteristics between arithmetic and algebra lies in the fact that arithmetic involves the direct computation of known values, whereas algebra necessitates inference and manipulation of unknown quantities or variables. As shown in Figure 2, students with arithmetic thinking scored below 1.5 on GA1-GA6, indicating their limited capacity to operate solely on known quantities and their inability to discern the structural aspects of expressions. The responses to GA1 and GA2, as exemplified in Figure 3, revealed that these students could only add both sides of the equal sign or calculate specific values of unknown variables to establish equality, thus demonstrating computational thinking.

The average scores on QR8–QR10 were all below 0.3, indicating their inability to complete quantitative reasoning tasks involving variables. In the functional thinking tasks (FT7 and FT12), their average scores reached approximately 1 point, suggesting that they were capable of employing recursive thinking to determine the next term but were unable to generalize this approach to distant terms. For instance, in FT12 (Figure 1), they could successfully solve the first question but encountered difficulty when attempting to determine the 20th term.

#### 4.2.2. Concrete Algebraic Thinking Students

**Figure 4:**
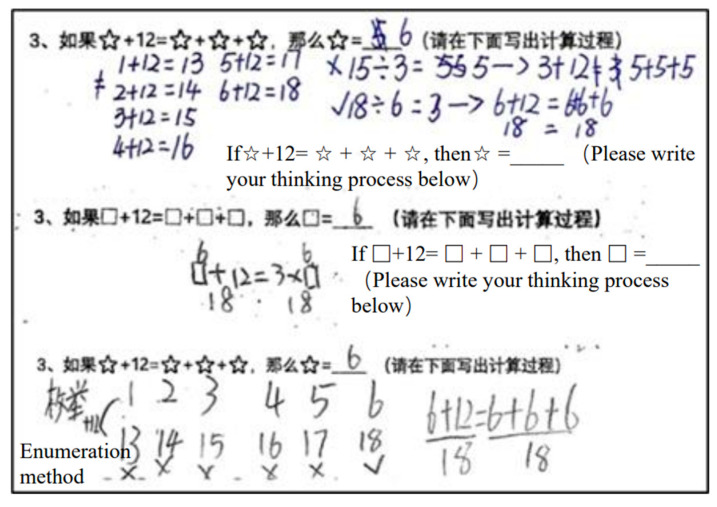
Three examples of commonly employed strategies employed by students with concrete algebraic thinking.

Compared to students with “arithmetic thinking”, students with “concrete algebraic thinking” demonstrated an ability to acknowledge the presence of “unknown” and were capable of identifying the implicit structure and patterns within equations, as reflected in their average scores on generalized arithmetic tasks, which hovered around 2 points (Figure 2). Students in concrete algebraic thinking had the following characteristics:

First, they could determine equality relationally. Relational thinking implies that students can discern equality by observing the relationships between numbers in an equation without the need for explicit numerical computation. For example, in the GA1 task, students with concrete algebraic thinking would provide an equation such as 58 + 65 = (57 + 1) + (66 − 1); second, they were able to use the “guess and check” strategy to find the value of the unknown quantity (e.g., Figure 4). Third, they could identify covariation relationships, such as “Every time you add a desk, you add 3 more people”. However, they could only perform calculations on specific terms and could not generalize to distant terms. Generalizing the patterns in function tasks and solving for “distant terms” places greater demands on students’ algebraic thinking, which can be challenging for those with concrete algebraic thinking.

**Figure 5:**
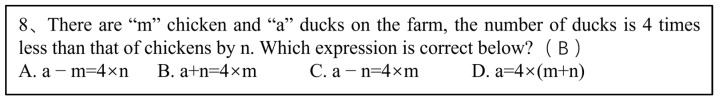
The quantitative reasoning Task QR8.

Similarly, while these students could employ a “guess and check” method to solve for unknown variables in equations, they might still struggle to solve similar equations involving unknowns when the equation’s structure became overly complex or when the values of the unknowns could not be easily guessed. In addition, students at this cognitive stage also found it challenging to comprehend the representation of quantity relationships and variables using letters, such as in QR8 (in Figure 5).

Consequently, they generally encountered difficulties in solving problems related to quantitative reasoning. Below is an interview with one of the students exhibiting “concrete algebraic thinking” (R represents the interviewer, and S represents the interviewed student):

1 R: Do you have any idea about Item 8 (in Figure 5)?
2 S: I couldn’t figure it out.
3 R: Is it because the questions involve variables that you encounter difficulties?
4 S: I usually tend to calculate specific numerical values… (the student re-read the question) So, what is this question asking for?
5 R: So, if there are no specific numbers, you wouldn’t know what it’s asking? If we substitute the letters with actual numbers, do you have an idea?
6 S: If m equals 10… (student started to draw the number line), then The number of chicks represents one unit, and the number of ducks is four times fewer, let’s say it’s 2 fewer. So, the ducks are 10 + 2 = 12, and 12 × 4 = 48 in total.
7 R: So, if substitute it with specific numbers, you know how to calculate, but you’re not sure with the letters?
8 S: If it’s with letters, I don’t understand what the question is asking.
9 R: If the number of chicks is m, and ducks are n, then ducks n = 4 × m − 2. Do you understand this equation? Do you think this equation is correct?
10 S: Firstly, you can move the 2 to the left side of the equation; I also don’t know why it can be done this way.
11 R: You can understand that this equation is valid, but not being able to solve for this letter makes you feel quite frustrated, right?
12 S: Of course, it’s really annoying when you can’t figure out the actual numbers.

From the interview presented above, it can be observed that students at the level of concrete algebraic thinking heavily rely on “specific numerical values”. They are accustomed to performing operations on known quantities, as described in lines 4 and 8 of the interviews, and are unable to proceed when encountering unknowns. They tend to substitute “specific numerical values” into unknowns to validate the correctness of the problem. Therefore, whether it is the “guess and check method” in GA3 or attempting to substitute specific numerical values into letters in QR8 (as shown in line 6 of the interview), both reflect this mode of thinking.

Overall, students with concrete algebraic thinking appeared to use relational thinking in generalized arithmetic tasks and find covariation relationships in functional thinking tasks. However, they could only deal with specific problems without generalization and thus could not solve the quantitative relation tasks with variable notations to the same extent that students with arithmetic thinking could.

#### 4.2.3. Generalized Algebraic Thinking Students

Compared to students with concrete algebraic thinking, those with generalized algebraic thinking exhibited a significant improvement in generalization. They no longer solved problems solely through enumeration but were capable of deducing general patterns and structures. In generalized arithmetic, they could use inverse operation or equivalence property to find the missing quantity value, such as removing one pentagon on both sides or “transposing”. Furthermore, they could find the general rules or relationships but not represent them accurately using variables.

**Figure 6:**
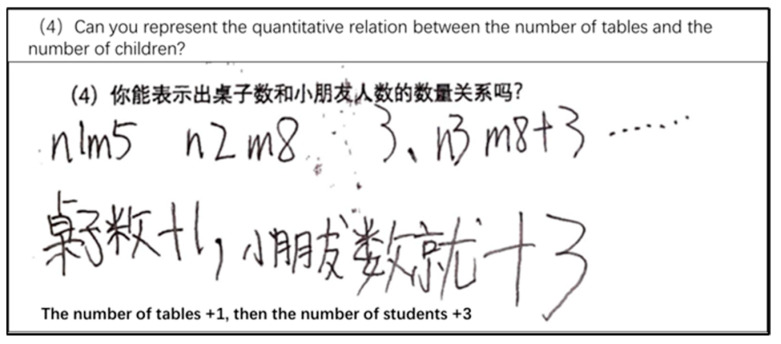
Generalized algebraic thinking students’ answering strategy for Question 12 (4).

In functional thinking tasks, students with generalized algebraic thinking could generalize the covariation relationship between the number of tables and the number of people and use literal language to represent the rule. However, they were not able to understand the variables correctly. For example, Figure 6 depicts the solution strategies of generalized algebraic thinking students on Task FT12 (Figure 1). In Question (4), the letters “n” and “m” were used to indicate the names of tables and people rather than variable notation. Although students were familiar with letters, they struggled to accurately express quantitative relationships using them. Taking Figure 6 as an example, students used “ellipsis” to signify the generalization of patterns. During interviews with such students, deviations in their understanding of the letter “n” also become evident.

1 R: How did you figure out how many people should be at Table 20?
2 S: I looked at the chart and saw that the number goes up by 3 each time. So I kept adding 3 and got to 62!
3 R: So what if it’s the nth table? Do you get what I mean?
4 S: Does that mean like endless tables?
5 R: If I tell you any table number, can you figure out how many kids are there?
6 S: If it’s a lot of tables, and I keep adding 3, I won’t know how many people there are. Is it n people?
7 R: Why is it n people?
8 S: Because n tables means a lot of people, and endless tables mean endless people!
9 R: ”So, who do you think is bigger, n or “n − 1”?
10 S: If a number is super big, like endless, then you can’t really compare it anymore.

Based on interviews, it is evident that the conceptualization of letters as numbers presents a cognitive challenge for elementary school students, particularly when these letters are used to represent variables. Through assessments and interviews, it was observed that many elementary school students equated the letter “n” with “infinity.” This misconception contributes to negative transfer in their understanding of variables represented by letters, such as their belief that “n” and “n − 1” are incomparable.

In tasks involving quantitative reasoning, students with generalized algebraic thinking were able to substitute abstract symbols in the problems with concrete numerical values. At the level of generalized algebraic thinking, students are more accustomed to expressing patterns through natural language or graphical representations, rather than using alphabetical symbols. Therefore, students at this cognitive level are already quite proficient at completing tasks in the test, although they still lack proficiency in aspects such as “representing variables with letters” and “proving statements using formal mathematical symbols”. In our sample for this study, the largest proportion of students, constituting 41.8%, were at this level of thinking.

#### 4.2.4. Symbolic Algebraic Thinking Students

Students classified under Group 4 demonstrated the capability to employ formal mathematical notation for reasoning, particularly in understanding functional relationships, quantitative relations, and the properties of operations. Such cognitive abilities are indicative of their well-developed, conventional algebraic thinking. In tasks involving generalized arithmetic, students with symbolic algebraic thinking were capable not only of employing “relational thinking” and “inverse operations” to solve equations but also of utilizing formal symbols for algebraic reasoning. For instance, in GA1, the responses of students with symbolic algebraic reasoning were not limited to identifying problem-specific patterns, such as “58 is one more than 57, and 85 is one less than 86”. They were also able to employ symbols to generalize these patterns into a more universal form, like “a + b= (a − 1) + (b + 1) = (a + 1) + (b − 1)”. Furthermore, students with symbolic algebraic thinking were also capable of reasoning and providing proofs for the generalized patterns they had abstracted.

In tasks focused on functional thinking, students with symbolic algebraic reasoning skills could discern the correspondence relationship between independent and dependent variables. They were also able to encapsulate these relationships in generalized symbolic forms, such as m = 3n + 2 for finding the number of children (m) as a function of the number of tables (n), or y = 5x − 2(x − 1) for finding the number of children (y) in terms of the number of tables (x). Beyond this, these students were capable of using alphabetic symbols to generalize quantitative relationships and understand that these symbols represent variables. In tasks involving quantitative reasoning, even though some students might still require supplementary representations like number lines, they were able to directly engage in algebraic reasoning with the abstract symbols presented in the problems. For example, in quantitative reasoning tasks, students could understand the letter “n” as a variable and use the letter to represent the quantitative relationship.

Table 3 presents a detailed performance analysis of students with the four different types of thinking in tasks related to generalized arithmetic, functional thinking, and quantitative reasoning.

In summary, arithmetic thinking students could only calculate the specific numerical value but could not calculate the unknown quantity. At the same time, they could not understand that letters represent numbers and variables. Students with concrete algebraic thinking could solve problems with specific values but without generalization. Those with generalized algebraic thinking could generalize the properties of operations and functional relationships, although their representation was not as accurate as when using formal symbolic notation. Those with symbolic algebraic thinking were able to use variable notation to generalize and represent the functional and quantitative relationships.

### 4.3. The Developmental Trend in Early Algebraic Thinking

**Figure 7:**
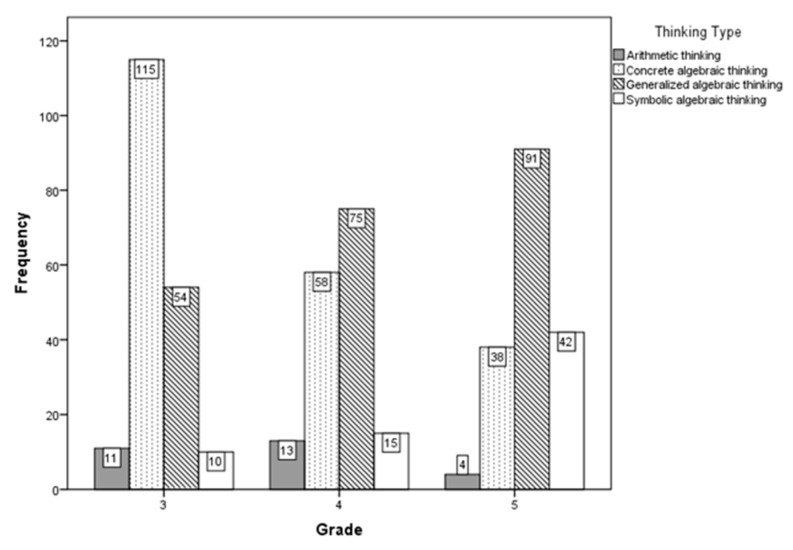
Frequency statistics of algebraic thinking levels among students of different grades.

The development of students’ mathematical thinking exhibited a stage-wise progression. An analysis of the types of thinking across different grade levels can assist us in understanding the developmental trajectory of students’ early algebraic reasoning. Figure 7 presents the frequency distribution of cognitive levels among students across the various grades.

The analysis of Figure 7 reveals that among third-grade students, those with concrete algebraic thinking constitute the largest proportion. However, as the grade level increased, the percentage of students with concrete algebraic thinking significantly declined, falling from 60.5% in the third grade to 36.0% in the fourth grade, and further to 21.7% in the fifth grade. Conversely, the proportion of students with generalized algebraic thinking gradually increased, becoming the majority in both fourth and fifth grades, at 46.6% and 52%, respectively. This indicates that as students advance through grades, their reliance on concrete numerical values decreases, and they become more capable of using relational thinking and inverse operations to solve problems involving unknowns. The proportion of students with symbolic algebraic thinking remained relatively low in both third and fourth grades, at 5.3% and 9.3%, respectively, suggesting that the ability to understand variables through alphabetic symbols and to directly engage in algebraic reasoning with these symbols is still underdeveloped at these stages. Even by fifth grade, the proportion of students with symbolic algebraic thinking remained substantially lower than those with generalized algebraic thinking.

## 5. Discussion

The development of algebraic thinking is of great significance to students’ mathematical ability, and it has gradually become the consensus of mathematics educators to develop students’ algebraic thinking from elementary school (Carpenter et al. 2003; Carraher et al. 2006). To gain deeper insights into the development of early algebraic thinking among elementary school students, latent class analysis (LCA) was conducted on responses from 526 students across Grades 3–5 in Shanghai. The results indicate that, based on the performance in the answers, students can be categorized into four types. Through the qualitative analysis of the answering strategies of students in the same category, different characteristics were observed in the early algebraic thinking of these four types of students on three kinds of mathematical tasks: generalized arithmetic, functional thinking, and quantitative reasoning. These are, respectively, denoted as arithmetic thinking, concrete algebraic thinking, generalized algebraic thinking, and symbolic algebraic thinking.

Generally, students characterized by arithmetical thinking are limited to solving mathematical tasks involving specific numbers. This limitation is evident in their approach to determining equality, which relies solely on specific calculations, rendering them entirely incapable of solving mathematical tasks involving variables. Students with concrete algebraic thinking can address problems involving unknowns through a “guess and check” method, but their capacity for generalization remains limited. This is manifested in their ability to enumerate only a restricted number of cases without achieving generalization. Students exhibiting generalized algebraic thinking can tackle mathematical tasks involving unknowns by utilizing inverse operations or fundamental properties of equations. They can discern generalized laws and represent them informally, such as through natural language. Finally, students with symbolic algebraic thinking can reason and demonstrate the structure of equations and the rule of change with formal mathematical symbols.

Through a comparative analysis of students embodying the four types of algebraic thinking, it becomes apparent that students’ thinking evolves from arithmetic thinking to concrete algebraic thinking, advances to generalized algebraic thinking, and ultimately culminates in symbolic algebraic thinking. In terms of the completion of mathematical content, this study aligns with previous research (Chimoni et al. 2018), demonstrating a consistent pattern in students’ capability to complete various mathematical tasks. Students find tasks of generalized arithmetic to be the most manageable, followed by tasks requiring functional thinking, and lastly, those necessitating quantitative reasoning. The distinction lies in that, in this study, even students with arithmetical thinking managed to identify recursive patterns in tasks involving functional thinking. In contrast, in the study by Blanton et al. (2015a), students of lower ability could only accomplish portions of the generalized arithmetic task and were unable to detect any patterns of change.

Concerning the evolution of specific concepts, the outcomes of this investigation are fundamentally in agreement with those of previous research. Regarding the comprehension of the equals sign and the progression of relational thinking, numerous studies have discerned that students’ relational thinking unfolds along a trajectory from “rigid operational thinking” to “computational thinking”, culminating in “relational thinking” (Rittle-Johnson et al. 2011; Blanton 2013). The findings of this study reveal that, although instances of rigid operational thinking are scarce in the samples, students do exhibit a progressive development from arithmetic thinking to relational thinking. This might be associated with the integration of concepts of equality and inverse operations in Chinese textbooks from early grades (Cai et al. 2011; Cai 2004b). Concerning the developmental trajectory of functional thinking, Chinese students exhibit a progression characterized by “recursive thinking–covariant thinking–corresponding thinking”, a trend in concordance with the research findings from other nations (Stephens et al. 2017b; Blanton et al. 2015b). Furthermore, the way students employ letters to symbolize numbers also adheres to a comparable developmental law. Numerous students employed “the number of tables + 1 = the number of people + 3” to articulate the discerned function covariation law, wherein the use of letter representation implied that students perceived letters as placeholders for “a fixed but arbitrarily selected number”. As students acquire a more profound comprehension of variable representation through letters, they transition to using representations such as “y = 3x + 2”, indicative of a deeper understanding of the role of letters in representation (Blanton et al. 2017).

The evolution from arithmetic thinking to symbolic algebraic thinking is marked by the enhancement of two pivotal capabilities: the ability to generalize and the ability to symbolize. A defining characteristic of algebraic thinking is “the capacity to formulate generalizations and articulate universal conclusions through progressively formal and conventional symbolic systems” (Kaput 2008). While symbolic manipulations hold significance in algebra, the essence of early algebraic thinking is the formation of generalizations. The capacity for generalization, nurtured during arithmetic learning, lays a robust foundation for subsequent explorations in algebra. Through the analysis of students’ responses, it is discerned that students with more advanced development in algebraic thinking are increasingly likely to opt for formal algebraic symbols for representation. Although many early algebra researchers do not heavily emphasize the necessity for students to employ formal algebraic symbols for inferential reasoning (Radford 2014), the cultivation of early algebraic thinking should prioritize the enhancement of students’ representational capabilities. Students should be encouraged to represent in their own ways, for instance, utilizing tables, line graphs, diagrams, etc., to depict patterns and relationships (Deborah Schifter 2009). When students have achieved higher levels of generalization ability, the use of algebraic symbols serves merely as an aid to conduct algebraic reasoning with greater accuracy and conciseness.

Based on the preceding discussion, we now provide some suggestions to help teachers better nurture early algebraic thinking in students. Firstly, it is crucial to enhance students’ understanding of how letters represent numbers during their elementary school years. Our research shows that students often struggle to grasp that letters can stand for both general numbers and variables. This is closely connected to the way we teach. Many teachers often use “letters representing numbers” as the foundation for teaching equations, focusing on their role as “unknowns” and overlooking their role as “general numbers” or “variables” (Ursini and Trigueros 2001). Secondly, we suggest introducing graphical pattern tasks into the curriculum starting from early grades to develop students’ functional thinking. These math tasks typically involve concrete shapes like circles or squares. Teachers can use various teaching tools to help students discover patterns. Beginning with identifying recurring changes between initial and later items, gradually introducing function tables can assist students in recognizing relationships and correspondences between variables.

## 6. Limitations and Implications

This study puts forward four stages of the early algebraic thinking development of primary school students through cross-sectional data. There are some limitations. Firstly, the results of the study are related to the sample to a certain extent. Students’ cognitive development is closely related to school education. The sample surveyed in this study included students from Shanghai, China; hence, the results of the study are more applicable to students in similar courses and textbook systems. A larger sample from a wider area is needed. At the same time, lower-grade or junior high school students can be included in the sample to explore the development of algebraic thinking in the transition stage from elementary school to junior high school. Secondly, there are limitations in inferring the developmental trajectory of students’ thinking through cross-sectional data; hence, future studies could employ longitudinal research to further validate the conclusions. Moreover, this study, based on latent class analysis, identified four groups and then delineated the developmental progression from arithmetic thinking to symbolic algebraic thinking through a qualitative analysis of the characteristics of each group. Although the four groups of students identified through quantitative analysis shared similar cognitive characteristics, there was still the possibility of individual students displaying different behaviors on certain items.

There are some aspects that we can explore further in the future. Firstly, we can include middle school students in our research sample. Early algebra courses aim to deepen arithmetic concepts and lay the foundation for students’ future algebra learning. Therefore, conducting longitudinal studies to understand the transition of students’ thinking from elementary to middle school is of significant value. Secondly, we can investigate teachers’ beliefs about early algebra and how their teaching practices impact students’ early algebraic thinking. Currently, most research in early algebra focuses on curriculum development and student surveys. However, existing studies have shown that teachers’ understanding of curricula and their beliefs can have a significant impact on students’ cognitive development (Cai 2004b). To effectively implement early algebra concepts in the classroom, it is crucial to ensure that teachers have a deep understanding of these concepts. Therefore, efforts should be made to integrate early algebra concepts into the professional development of elementary school teachers, enriching their understanding of elementary arithmetic courses.

## Figures and Tables

**Table 1:** Description of the items in the algebraic thinking test.

Content Strand	Concept	Task
Generalized arithmetic (GA)	Equality, Equal sign, Equivalence, Equations	GA: 1, 2, 3, 4
	Simple operation	GA: 5
	Properties of operations	GA: 6
Functional thinking (FT)	Letter as variable	FT: 11
	Covariable and correspondence	FT: 7, 12
Quantitative reasoning (QR)	Generalizing the quantitative relationship	QR: 8, 9, 10

Note: GA, FT, and GR are the abbreviations of generalized arithmetic, functional thinking, and quantitative reasoning.

**Table 2:** Comparison of latent class analysis model fit.

Model	K	Log(L)	AIC	BIC	aBIC	Entropy	LMR	BLRT
1 Class	31	−6230.14	12,522.28	12,654.51	12,556.11	—	—	—
2 Class	63	−5720.43	11,566.86	11,835.57	11,635.60	0.82	**	**
3 Class	95	−5618.78	11,427.56	11,832.77	11,531.21	0.78	0.52	**
4 Class	127	−5516.74	11,377.47	11,919.17	11,516.04	0.83	0.59	**
5 Class	159	−5518.23	11,354.47	12,032.65	11,527.94	0.82	0.79	**
6 Class	191	−5483.39	11,348.77	12,163.44	11,557.16	0.85	0.79	1.00

Note: ** *p* < .01.

**Table 3:** Performance of students with different thinking types in mathematical content.

Thinking Type	Generalized Arithmetic	Functional Thinking	Quantitative Reasoning
Arithmetic thinking	Able to judge the equality of equations by calculation. Can only perform calculations on known specific numbers and cannot perform calculations on unknown quantities. Able to identify the existing laws within calculations and to extend examples but unable to generalize.	Able to find recursive rules in patterns (e.g., the number of people increased by 3), but cannot make a generalization. Cannot understand the letters to represent variables.	Inability to understand mathematical problems without specific numbers.
Concrete algebraic thinking	Able to use relational thinking to determine the equality of an equation. Able to solve the unknowns in the equation by “guess and check”. Able to identify patterns within calculations and extend examples, but unable to generalize them.	Able to discern covariant rules in patterns (e.g., For every additional desk, three more people are added.), but unable to generalize. Cannot understand the letters to represent variables.	Inability to understand mathematical problems without specific numbers.
Generalized algebraic thinking	Able to use relational thinking to determine the equality of an equation. Can solve for the unknown in the equation directly through inverse operations or the basic properties of the equation. Able to identify patterns in operations and extend them to generalizations but unable to use formal symbols for a comprehensive demonstration.	Able to discern covariant rules in patterns (e.g., For every additional desk, three more people are added.), but unable to generalize. Cannot understand the letters to represent variables.	Able to employ specific numerical values in place of abstract quantities in problems, or to use line graphs for quantitative reasoning.
Symbolic algebraic thinking	Able to use relational thinking to determine the equality of an equation. The unknown quantity in the equation can be solved directly through the inverse operation or the basic properties of the equation. Able to discover the laws in operations and generalize them and use formal symbols to represent and demonstrate correctly.	Able to use covariation thinking or correspondence thinking to find functional relationships and use formal symbols to correctly express general terms. Able to understand that letters represent variables and can correctly represent the correspondence in functional tasks using algebraic symbols.	Able to directly conduct symbolic algebraic reasoning on the quantitative relationships in mathematical situations.

## Data Availability

The datasets used and/or analyzed during the current study are available from the corresponding author upon reasonable request.
